# Resting-State Functional Connectivity Bias of Middle Temporal Gyrus and Caudate with Altered Gray Matter Volume in Major Depression

**DOI:** 10.1371/journal.pone.0045263

**Published:** 2012-09-24

**Authors:** Chaoqiong Ma, Jurong Ding, Jun Li, Wenbin Guo, Zhiliang Long, Feng Liu, Qing Gao, Ling Zeng, Jingping Zhao, Huafu Chen

**Affiliations:** 1 Key Laboratory for NeuroInformation of Ministry of Education, School of Life Science and Technology, University of Electronic Science and Technology of China, Chengdu, PR China; 2 Mental Health Institute, The Second Xiangya Hospital, Central South University Changsha, Hunan, China; 3 Mental Health Center, First Affiliated Hospital, Guangxi Medical University, Nanning, Guangxi, China; Institution of Automation, CAS, China

## Abstract

Magnetic resonance imaging (MRI) studies have indicated that the structure deficits and resting-state functional connectivity (FC) imbalances in cortico-limbic circuitry might underline the pathophysiology of MDD. Using structure and functional MRI, our aim is to investigate gray matter abnormalities in patients with treatment-resistant depression (TRD) and treatment-responsive depression (TSD), and test whether the altered gray matter is associated with altered FC. Voxel-based morphometry was used to investigate the regions with gray matter abnormality and FC analysis was further conducted between each gray matter abnormal region and the remaining voxels in the brain. Using one-way analysis of variance, we found significant gray matter abnormalities in the right middle temporal cortex (MTG) and bilateral caudate among the TRD, TSD and healthy controls. For the FC of the right MTG, we found that both the patients with TRD and TSD showed altered connectivity mainly in the default-mode network (DMN). For the FC of the right caudate, both patient groups showed altered connectivity in the frontal regions. Our results revealed the gray matter reduction of right MTG and bilateral caudate, and disrupted functional connection to widely distributed circuitry in DMN and frontal regions, respectively. These results suggest that the abnormal DMN and reward circuit activity might be biomarkers of depression trait.

## Introduction

Major depressive disorder (MDD), one of the most common psychiatric disorders, ranks among the top causes of disability and worldwide disease burden [1]. Clinically, patients with MDD present with a number of psychological and psychiatric symptoms characterized by multiple self-abnormalities, such as pervasive feelings of sadness, guilt, and worthlessness [2]. It is estimated that at any time as many as 5% of the population suffers from depression, and the prevalence of depression is increasing [3], [4]. In spite of current available effective treatments, it is consistently found that about 30–40% of patients with MDD fail to respond to antidepressants [4]. Non-responders are described as having treatment-resistant depression(TRD), while those, who respond to the antidepressants, are referred to treatment-responsive depression(TSD) [5]. Advances in imaging techniques such as positron emission tomography (PET), single photon computed tomography (SPECT) and functional magnetic resonance imaging (fMRI) make it feasible to understand the neuropathology of MDD [6]. However, the underlying etiology and pathophysiology of MDD are still not entirely understood.

Recently, voxel-based morphometry (VBM), a non-biased and fully automated whole-brain measurement technique, has been employed by numerous investigators [7]. Many previous studies have consistently found gray matter abnormalities including temporal lobe, basal ganglia, amygdala, hippocampus and orbitofrontal cortex (OFC) in MDD (see review [8]). Among these gray matter abnormalities, gray matter volume reduction in temporal lobe regions, especially in superior temporal gyrus, was consistently detected in a handful of MDD studies [8]. More recently, reduced gray matter volume in the bilateral MTG was also reported [9]. In addition, the caudate, a basal ganglia structure, is known to be involved in the control of motor, cognitive, and emotional processes. Using a voxel-based analysis, Shah et al. [10] showed that TRD patients had less caudate gray matter volume than recovered patients and healthy controls, suggesting that the structure deficits of caudate might lead to some clinical symptoms observed in MDD.

Evidences have increasingly shown that the production of emotions is unlikely to be the result of a single abnormal brain region or neurotransmitter system. Instead, it could be conceptualized as a distributed neuronal brain network consisting of cortical and limbic regions [11]. Therefore, brain abnormalities in MDD are much more likely to be present in functional connectivity (FC) between brain regions, rather than within discrete brain regions [12], [13]. FC has been defined as “the temporal correlation of a neurophysiological index measured in different areas” [14]. Studies on FC in patients with major depression have achieved varied results. Increased FC among the amygdala, hippocampus, and caudate-putamen regions during emotion processing [15] while reduced amygdala-prefrontal connectivity [16] have been reported during a facial expression processing task. The fMRI data has also elucidated the imbalance of OFC connectivity [17]. Additionally, Vasic et al. showed that the connectivity between subgenual cingulate and gyrus cinguli was disrupted during a verbal working memory task in MDD [18].

Although task-based fMRI studies can assess disturbances in FC, assessment of resting-state connectivity may have several potential advantages over task-activation fMRI in terms of its clinical applicability, for instance, it is difficult for some sick patients to perform a task correctly [19]. Several recent fMRI studies have found decreased FC in the cortico-limbic circuit [13], [20] and increased FC within the default-mode network (DMN) [21] in MDD during rest. Lui et al. suggested that patients with TRD were associated with disrupted FC mainly in thalamo-cortical circuits, while patients with TSD were associated with decreased connectivity in the limbic-striatal-pallidal-thalamic circuit during resting state [22]. In addition, the FC of hate circuit was reported in both first-episode MDD and TRD [23]. However, it is unclear whether the connectivity alterations are related to gray matter deficits within brain networks in MDD.

Along these lines, the main objective of this study is to investigate 1) whether gray matter abnormalities exist in patients with TRD and TSD and 2) whether the altered gray matter is associated with altered FC. Here, we for the first time use VBM and resting-state FC to perform a comprehensive evaluation of the neural circuitry underlying MDD.

## Materials and Methods

### Participants

Eighteen right-handed patients with TRD and 17 right-handed first-episode TSD patients were originally recruited from the Institute of Mental Health, the Second Xiangya Hospital of Central South University, China (**Table 1**). Major depression was diagnosed by two qualified psychiatrists (Dr Zhao J and Dr Liu Z) using the Structured Clinical Interview according to the DSM-IV criteria [24]. Exclusion criteria included bipolar disorder, any history of major illness, cardiovascular disease, and younger than 18 years or older than 50 years. An additional exclusion criterion for TSD patients was that the current illness duration was no more than six months. The severity of depression was assessed using the 17-item Hamilton Rating Scale for Depression [25] and only patients who scored 18 or greater were eligible for the study. Patients with TRD were taking at least two classes of antidepressants before participating in the study and treatment resistance was defined as non-responsiveness to at least two adequate trials (in terms of dosage, duration (6 weeks for each trail), and compliance) of different classes of antidepressants in consistent with previous studies [10], [26]. This non-responsiveness was defined as less than 50% reduction in HRSD score [27] after treatment at a minimum dose of 150 mg/day of imipramine equivalents [28] (dose converted using a conversion table) for 6 weeks. The TSD patients were at their first episode of MDD and treatment-naive. After fMRI scanning, all patients were directed to take antidepressants at a minimum dose of 150 mg/day of imipramine equivalents [28] (dose converted using a conversion table) for 6 weeks by two qualified psychiatrists (Dr Zhao J and Dr Liu Z). The drugs included one of the three typical classes of antidepressants: tricyclic antidepressants (TCAs), selective serotonin reuptake inhibitor (SSRIs) and serotonin-norepinephrine reuptake inhibitor (SNRIs). The treatment response was defined as a more than 50% reduction in the HRSD score after the antidepressant treatment, consistent with previous studies [10], [26], [27], [29]. Seventeen right-handed healthy controls were recruited from the community and had no history of neuropsychiatric illness or brain. Clinical and demographic data from all the 52 participants are shown in **Table 1**. The three groups were well matched for age, gender and years of education. All subjects were given information about the procedures and gave written informed consent via forms approved by the Ethics Committee of the Second Xiangya Hospital, Central South University.

**Table 1:** Demographic and clinical characteristics of TRD patients, TSD patients and healthy controls.

Variables(Mean±SD)	TRD	TSD	HC	P
Gender (M/F)	11/7	10/7	10/7	0.987^a^
Age (years)	27.39±7.74	26.71±7.73	24.24±4.41	0.368^b^
Education (years)	13.56±3.60	12.35±2.12	13.82±2.38	0.271^b^
Course (months)	35.5±49.89	2.59±1.33	-	0.010^c^
HAMD	23.89±3.69	25.58±6.32	-	0.335^c^

Abbreviations: TRD, treatment-resistant depression; TSD, treatment-responsive depression; HC, healthy controls; HAMD, Hamilton Depression Rating Scale.

a The P value for gender distribution in the three groups was obtained by chi-square test.

b The P values were obtained by one-way analysis of variance tests.

c Two sample *t*-test.

### Data Acquisition

Structure and functional imaging was performed on a 1.5T GE scanner (General Electric, Fairfield, Connecticut, USA) equipped with high-speed gradients. The participants were asked to use a prototype quadrature birdcage head coil fitted with foam padding to minimize head movement. They were informed to remain motionless, keep their eyes closed and not think of anything in particular. Axial anatomical images were acquired using a volumetric three-dimensional Spoiled Gradient Recalled sequence (SPGR) with the following parameters: repetition time/echo time (TR/TE) = 12.1/4.2 ms, 172 axial slices, 512×512 matrix, 15^0^ flip angle, 24 cm field of view (FOV), 1.8 mm section thickness and 0.9 mm gap. At the same locations to anatomical slices, functional images were acquired by using an echo-planar imaging sequence with the following parameters: TR/TE = 2000/40 ms, 20 slices, 64×64 matrix, 90^0^ flip angle, 24 cm FOV, 5 mm section thickness and 1 mm gap. For each participant, the fMRI scanning lasted for 6 min and 180 volumes were obtained.

### Voxel-Based Morphometry Analysis

Voxel-based morphometry analysis [30] was performed in SPM8 (http://www.fil.ion.ucl.ac.uk/spm). First, all T1-weighted anatomical images were manually reoriented to place the anterior commissure at the origin of the three-dimensional Montreal Neurological Institute (MNI) space. The images were then segmented into gray matter, white matter, and cerebrospinal fluid (CSF) [31]. A diffeomorphic non-linear registration algorithm (diffeomorphic anatomical registration through exponentiated lie algebra—DARTEL) [32] was used to spatially normalize the segmented images. This procedure generated a template for a group of individuals. The resulting images were spatially normalized into the MNI space using affine spatial normalization. An additional processing step consisted of multiplying each spatially normalized gray matter image by its relative volume before and after normalization. This ensured that the total amount of gray matter in each voxel was preserved. Finally, the resulting gray matter images were smoothed with an 8 mm full-width half-maximum (FWHM) isotropic Gaussian kernel.

Voxel-wise comparisons of gray matter volume between the three groups were performed using a one-way analysis of variance (ANOVA) followed by post-hoc *t*-test. Age and gender were modeled as covariates of no interest. The statistical significance of group differences in each region was set at p<0.005(AlphaSim corrected and minimum cluster size of 418 voxels), using the AlphaSim program in the REST toolkit (http://www.restfmri.net). Based on the previous studies, the caudate plays an important role in MDD and the volume alteration of this region has been reported. In order to detect whether the caudate atrophy exists in the present study, a looser p threshold was chosen (p<0.01, AlphaSim corrected and minimum cluster size of 624 voxels), The AlphaSim correction was conducted using the AlphaSim program in the REST software (http://www.restfmri.net), which applied Monte Carlo simulation to calculated the probability of false positive detection by taking both the individual voxel probability thresholding and cluster size into consideration [33]. To identify the effect of illness progression to the structure abnormalities, the average gray matter volume values for all the voxels in abnormal areas, revealed by voxel-based morphometry, were extracted and correlated with the duration of illness using correlation analysis.

### Functional Connectivity Analysis

The fMRI images were initially corrected for temporal differences and head motion. None of the depressive patients had more than 3 mm head motion and 3°of rotation during the whole fMRI scan. And the healthy controls were under 1 mm head motion and 1°of rotation. Each voxel was resampled to 3×3×3 mm^3^, applying the Montreal Neurological institute (MNI) echo-planer imaging template. Then, the images were spatially smoothed at 8 mm FWHM.

FC was investigated using a temporal correlation approach [34], [35]. Regions showing significantly altered gray matter volume were defined as seed ROIs for subsequent FC analysis. The time series of each ROI was preprocessed as follows: first, six head motion parameters, the averaged signals from CSF and white matter, and the global brain signal were regressed [34], [35]; second, the time series were band filtered (0.01–0.08 Hz) to reduce the effects of low-frequency drift and high frequency noise. The residuals signal was used as the regional time series of ROI for further analyses. A correlation analysis was conducted between the seed ROI and the remaining voxels in the whole brain. The resulting r values were converted using Fisher's r-to-z transformation to improve the Gaussianity of their distribution.

For each group and each seed ROI, individual z value maps were analyzed with a random effect one-sample *t*-test to identify voxels showing a significant positive correlation with the seed time course. The significance level for each group was set at p<0.005 using AlphaSim correction (with combination of threshold of p<0.005 and a minimum cluster size of 46 voxels).

To compare the FC maps between the three groups, one-way ANOVA followed by post-hoc *t*-tests was employed among the three groups (TSD vs. HC, TRD vs. HC, TRD vs. TSD). Age and gender were also modeled as covariates of no interests. The significance level of group level was set at p<0.005. The group comparison was restricted to the voxels with significant correlation maps of TRD patients, TSD patients and healthy controls, by using an explicit mask from the union set of the one-sample *t*-test results (p<0.005, AlphaSim corrected) of the three groups.

## Results

### Morphometry Analysis

**Figure 1:**
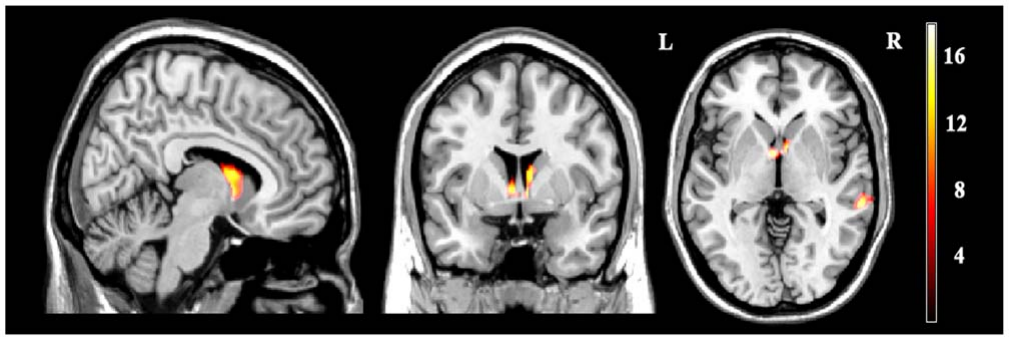
Statistical parametric images of voxle-based morphometry analysis among TRD,TSD and healthy controls. Significantly altered gray matter volume was detected in the right middle temporal gyrus and the bilateral caudate. Color scales represent T values using one-way ANOVA (p<0.005, AlphaSim corrected).

After one-way ANOVA, the significant gray matter deficits were found in two brain regions: right MTG (MNI coordinates: 61,-34,-3; voxel size = 516) and bilateral caudate (MNI coordinates:7,6,10; voxel size = 626) (**Figure 1**). Relative to healthy controls, both the TRD patients and TSD patients exhibited decreased gray matter volume in the right MTG while only TRD patients exhibited reduced gray matter volumes in the bilateral caudate. Compared to TSD patients, TRD patients exhibited reduced gray matter volumes in the bilateral caudate. Moreover, significant negative correlations were observed between the degree of gray matter volume reduction in the right MTG and the duration of illness in TSD patients (p = 0.02, r = −0.54).

Figure 1 shows statistical parametric images of voxle-based morphometry analysis among TRD, TSD and healthy controls. Significantly altered gray matter volume was detected in the right middle temporal gyrus and the bilateral caudate.

### Functional Connectivity Analysis

The significant gray matter deficits detected among the three groups were selected as the seed areas for FC analysis. When taking the right MTG as the seed areas, TSD patients showed increased connectivity in the right superior temporal gyrus and decreased connectivity in the right angular gyrus, rectus, precuneus, medial frontal gyrus and bilateral superior frontal gyrus relative to TRD patients; compared to healthy controls, TSD patients showed increased connectivity in the left supramarginal gyrus and decreased connectivity in the right angular gyrus and left precuneus and parahippocampal gyrus; compared to healthy controls, TRD patients exhibited increased connectivity in the right precuneus, middle temporal gyrus, bilateral superior frontal gyrus, left middle frontal gyrus, and decreased connectivity in the right cuneus.

**Figure 2:**
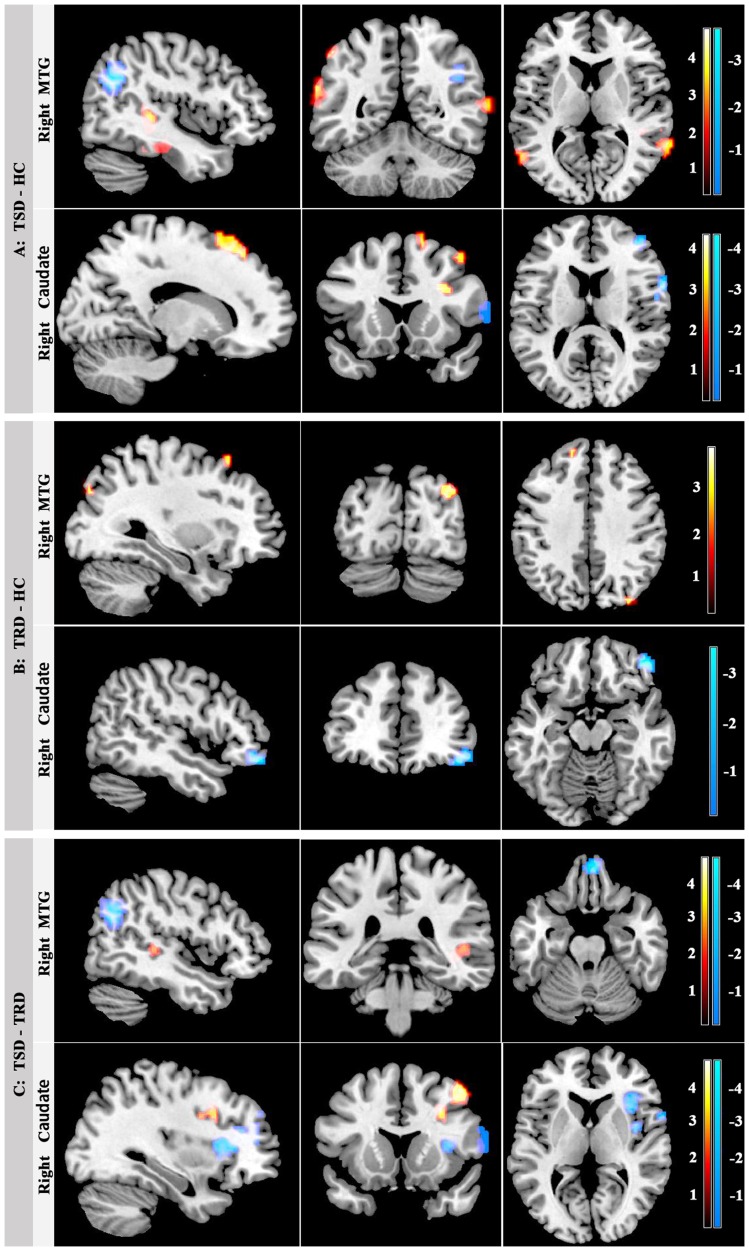
Aberrances in resting-state FC in patients with TSD and TRD and healthy controls. In panel A, abnormal connectivity with right MTG (top row) and right caudate (bottom row) in patients with TSD relative to healthy controls (HC); In panel B, abnormal connectivity with right MTG (top row) and right caudate (bottom row) in patients with TRD relative to healthy controls; In penal C, abnormal connectivity with right MTG (top row) and right caudate (bottom row) in TSD groups relative to patients with TRD. Color scales represent T values in each FC map using one-way ANOVA followed by post-hoc *t*-test. (p<0.005, AlphaSim corrected).

When the seed was located in the right caudate (the gray matter reduction in bilateral caudate was detected, but the main part of the cluster was in the right side), TRD patients showed increased connectivity in the right superior frontal gyrus and middle frontal gyrus, and decreased connectivity in the right inferior frontal gyrus and corpus callosum compared to TSD patients; compared to healthy controls, TSD patients showed increased connectivity in the right inferior frontal gyrus (opercula part), middle frontal gyrus and bilateral superior frontal gyrus, and decreased connectivity in the right middle frontal gyrus, inferior frontal gyrus (opercula part), insula and left middle occipital gyrus; compared to healthy controls, TRD patients exhibited decreased connectivity in the right middle OFC and left occipital gyrus. (**Figure 2** **,** **Table 2**). Figure 2 shows aberrances in resting-state FC in patients with TSD and TRD and healthy controls.

**Table 2:** Aberrances of functional connectivity in patients with TSD, TRD, and healthy controls.

Seed ROI	Connected region	Side	Cluster voxels	MNI(x,y,z)	T value
TSD patients-healthy controls					
Right MTG	Supramarginal gyrus	L	33	−63,−51,27	4.38
	Precuneus	L	278	−6,−66,15	−3.63
	Parahippocampal gyrus	L	67	−18,−39,9	−3.61
	Angular gyrus	R	160	42,−63,30	−4.72
Right Caudate	Inferior frontal gyrus (opercula)	R	77	30,15,30	4.83
	Superior frontal gyrus	L	99	−15,33,48	4.27
	Superior frontal gyrus	R	295	12,36,51	4.49
	Middle frontal gyrus	R	134	42,12,51	5.15
	Middle occipital gyrus	L	51	−27,−93.18	−4.88
	Middle frontal gyrus	R	74	36,45,12	−4.31
	Insula	R	33	39,−3,3	−3.41
	Inferior frontal gyrus (opercula)	R	171	63,9,9	−4.98
TRD patients-healthy controls					
Right MTG	Middle temporal gyrus	R	39	57,−33,−18	3.16
	Middle frontal gyrus	L	133	−42,21,30	3.70
	Superior frontal gyrus	L	168	−21,36,54	3.79
	Superior frontal gyrus	R	73	27,27,57	4.20
	Precuneus	R	41	3,−57,45	2.98
	Cuneus	R	126	18,−72,12	−3.51
Right Caudate	Middle orbitofrontal cortex	R	36	42,45,−15	−3.25
	Middle occipital gyrus	L	40	−33,−90,21	−3.81
TSD patients-TRD patients					
Right MTG	Superior temporal gyrus	R	109	48,−30,6	3.86
	Medial frontal gyrus	R	108	12,51,24	−3.25
	Angular gyrus	R	223	42,−60,27	−4.28
	Precuneus	R	172	3,−54,39	−4.13
	Superior frontal gyrus	L	90	−9,42,51	−3.41
	Superior frontal gyrus	R	41	36,21,54	−3.61
	Rectus	R	79	6,54,−24	−4.07
Right Caudate	Middle frontal gyrus	R	165	45,18,54	4.99
	Superior frontal gyrus	R	34	9,30,66	3.62
	Inferior frontal gyrus	R	160	33,30,15	−4.31
	Corpus callosum	L	46	−12,30,3	−3.84

Abbreviations: TRD, treatment-resistant depression; TSD, treatment-responsive depression.

## Discussion

To the best of our knowledge, this is the first study to combine structure MRI and resting-state functional MRI to investigate alterations within brain regions in patients with TRD and TSD. We found that both the patients with TRD and TSD showed significant gray matter abnormalities in right MTG and bilateral caudate. To further investigate the influence of structure changes to functional circuits, seed-based resting-state FC analysis was performed, and abnormal connectivity in right MTG-DMN and caudate-prefrontal circuit were shown in both patient groups. These findings demonstrated that the structure reduction of MTG and caudate, and, altered functional connection to widely distributed circuitry in DMN and prefrontal regions respectively might contribute to disturbances in mood and cognition in MDD patients.

### Altered gray matter and functional connectivity in right MTG

In the present research, reduced right MTG volume was found in both the patients with TRD and TSD compared to healthy controls. The MTG is located in the extended dorsal attention system and is involved in cued attention and working memory [36], [37]. Using the optimized VBM method, Peng et al. reported reduced gray matter volume in the bilateral MTG in a group of first-episode MDD [9]. Our result of reduced gray matter volume in the right MTG partly concurred with Peng et al. [9]. Particularly, in our study, the gray matter volume in the right MTG was negatively correlated with the course of disease in TRD patients, which might point to possible anatomical substrates of TRD expressed by volumetric abnormalities. In addition, Wu et al. [38] reported that TRD patients showed higher regional homogeneity in the right MTG than treatment non-resistant depression patients and healthy controls. Moreover, lower amplitude of low-frequency fluctuations values in this region was reduced in both the patients with TRD and TSD [39]. These findings may suggest that MTG are presumably part of a relevant functional network associated with MDD. To further investigate the influence of reduced MTG volume to functional circuits, resting-state FC analysis of MTG was performed.

Our results showed that TSD patients exhibited abnormal connectivity in supramarginal gyrus, parahippocampa gyrus, precuneus and angular gyrus, while TRD patients in precuneus, cuneus, middle frontal gyrus, middle temporal gyrus and superior frontal gyrus. Though these two subtypes of depression exhibited aberrant connectivity in different regions, most of these regions were located in the DMN. The DMN exhibits high levels of activity during resting state and decreases the activity for processes of externally oriented mental activity, induced by a wide range of sensory and cognitive tasks [40]. It has been shown to play a critical role in the neurophysiological processes of episodic memory, self-reflective and emotional regulation [41]. Moreover, DMN is commonly regarded as a key brain network in MDD, and abnormalities in DMN have been observed in many previous studies. In a recent resting-state fMRI study, Zhang et al. [42] used graph theory-based approaches and found that the MDD-related increases in nodal centralities within the DMN regions. In another study using independent component analysis, Zhu et al. [43] reported the increased FC in anterior medial regions of the resting-state DMN which associated with rumination, whereas decreased FC in posterior medial regions which associated with overgeneral autobiographical memory, suggesting that abnormal DMN activity might be an MDD trait. Moreover, DMN-related aberrances of FC have also been observed in other MDD studies, such as between the subgenual cingulate and thalamus [21] and within the DMN regions [41], [44]. The altered MTG-DMN connectivity in present study extends the previous studies of abnormal FC within DMN of patients with MDD by investigating the FC of the right MTG. Major depression is characterized by negative automatic thoughts about self, the world, and the future, we speculate that the altered connectivity between the right MTG and DMN may contribute to the negative thoughts and negative emotional experience in the TRD and TSD patients [41].

### Altered gray matter and functional connectivity in Caudate

Besides the right MTG, gray matter abnormality was also found in the caudate. Compared to healthy controls, only patients with TRD exhibited reduced gray matter volumes in the caudate. Also, gray matter volumes of the caudate were reduced in TRD patients relative to TSD patients. Though several studies have detected the caudate atrophy in MDD [10], [45], [46], the gray matter decrease in caudate is still a disputable conclusion for the failure to detect gray matter decrease in this region has also been reported. Confounds associated with illness chronicity, such as illness duration, number of episode, medication and treatment response to antidepressants, may have contributed to the inconsistency across studies. Our current finding that only the TRD patients showed decreased gray matter volume in the caudate was consistent with Shah et al. [10]. As Lorenzetti et al. [8] suggested, caudate may be particularly affected in more severe persistent subtypes of depression.

The caudate, the main subregion of striatum, is one of the central loci for reward-based behavioral learning and therefore intricately involved in pleasure and motivation [47]. To assess differences in the crosstalk among brain regions, FC maps were calculated using the caudate as the seed ROI. We observed that both the patients with TRD and TSD showed FC aberrances in frontal lobes. TRD patients exhibited decreased connectivity in right middle OFC. TSD patients exhibited abnormal connectivity mainly in right inferior frontal gyrus, middle frontal cortex, and superior frontal cortex, partly belonging to dorsolateral prefrontal cortex (DLPFC: BA 9/46). DLPFC is associated with cognitive control, which is thought to play an important role in self-referential processing in major depression [48]. Combining a mixed monetary incentive delay/memory task and fMRI, Staudinger et al. suggested that the DLPFC might modulate striatal reward encoding during reappraisal of reward anticipation [49]. The OFC is involved in sensory integration, reward processing, decision-making, reward prediction and subjective hedonic processing [50]. In a reversal-learning fMRI study by O'Doherty et al., the lateral area of the OFC was found to be more activated following a punishing outcome and showed a positive correlation between the size of the loss and the magnitude of signal; in contrast, the medial OFC was more activated following a rewarding outcome and showed a positive correlation between size of reward and magnitude of signal [51].

The caudate together with DLPFC and OFC, plays a central role in reward-related/motivation process, which have been broadly elucidated in a convergence of reward-related functional neuroimaging studies [52], [53], [54], [55], [56]. In a group of adolescents with MDD, using a reward-related guessing task, Forbes et al. [54] demonstrated less striatal response than healthy comparison adolescents during reward anticipation and reward outcome, but more response in dorsolateral and medial prefrontal cortex. In another reward decision-making task fMRI study, Forbes et al. [55] found that the young people with MDD exhibited less neural response than control participants in reward-related regions including caudate and OFC during the reward anticipation and outcome phase of reward processing. Particularly, in the anticipation phase, the depressive symptoms were associated with activation in inferior OFC while in the outcome phase associated with activation in caudate. Moreover, employing a Wheel of Fortune (WoF) task, Smoski et al. [56] found that relative to affectively healthy control adults, MDD participants showed decreased activation during reward anticipation in the right caudate but greater activation during reward selection and in response to non-winning feedback in OFC. Evidence for reward systems being central to the interactions between these pathologies is apparent in the comorbidity of major depression and nicotine addition documented by Cardenas et al. [57] as well as a general dysfunction of dopaminergic action found in a variety of mental diseases [58]. As to depression, the reward dysregulation is associated with anhedonia, supported by neuropsychological evidence for altered reward sensitivity in MDD [59], [60]. Anhedonia, the inability to experience pleasure in things normally rewarding, has been considered as a potential trait marker related to vulnerability to depression [61]. Using a probabilistic reward task, Pizzagalli et al. [59] demonstrated that MDD subjects showed significantly reduced anhedonia capacity compared to controls, indicating that MDD was characterized by an impaired tendency to modulate behavior as a function of prior reinforcements. The caudate-prefrontal connectivity in both the patients with TRD and TSD was supported by the prior research of caudate-prefrontal FC circuit in a group of healthy controls, which confirmed the resting-state FC between the caudate and frontal regions including OFC, DLPFC, ventral lateral prefrontal cortex, inferior frontal cortex [53]. Given the role of caudate, DLPFC and OFC in reward processing, our results of abnormal caudate-prefrontal connectivity indicated the dysregulation of reward mechanisms, which might result in lack of motivation and anhedonia often observed in MDD.

In the present study, reduced gray matter volume in right MTG was found in both the patients with TRD and TSD but the altered caudate volume was found only in TRD patients, suggesting that the gray matter reduction in right MTG might serve as signature of a depressive “trait”, while the gray matter reduction in caudate a depressive “state”. Therefore, we may classify the TRD patients from the MDD based on the gray matter volume in the caudate. Ongoing research would be needed to clarify our speculation. What's more, in order to explore the influence of structure changes to functional circuits, the right MTG and caudate were used as seeds for FC. It is noteworthy that the patterns of resting state FC for the two structure abnormal regions are different among the TRD, TSD and healthy controls. Thus, gray matter volume alterations in the two regions appear to change the integration of affected brain regions within corresponding functional networks, at least with regard to resting-state indices of FC.

Though there were a large number of direct FC differences between the two subtypes of patient groups, it must be emphasized that all the patients with TRD in the present study received at least two classes of antidepressants before taking part in the study, so some of the differences observed between the two groups could be due to drug effect. Although the antidepressants were not helpful to the disease, the effect of medication should not be ignored in interpreting the difference between the patients with TRD and healthy controls. Recent studies [20], [62] suggested that antidepressants treatment might reverse the pattern of activation and connectivity abnormalities in depression. Therefore, some of imbalance in connectivity observed in the two subtypes of depression might be partly associated with medication effects. To control this problem, future studies should focus on drug-free patients with the two subtypes.

Other limitations should be also noted in the current study. First, our study was limited by a relatively small sample size, consequently, our preliminary results should be confirmed in a larger sample of MDD patients and healthy controls in future studies. Second, the current study was limited by the heterogeneous pharmacological profiles. Patients were treated with one of three different classes of antidepressants, thus the same patients might exhibit treatment non-response to an antidepressant but treatment response to another. Therefore, this heterogeneity might limit the translational value of our findings. For this reason, future studies need to choose patients taking the drugs with the same pharmacological profile. Third, like other studies using resting-state fMRI, we could reduce but could not completely eliminate the effects of physiological noises such as respiratory and heart rhythm by using a relatively low sampling rate. However, it would be difficult to cover the whole brain by using a relatively short TR. In the future, a more rigorous approach should be applied to remove such physiological noises [39]. Finally, though the gray matter alterations in limbic system have been consistently observed, we failed to find gray matter changes in the limbic system in the present study. Based on the previous studies, there were also a certain amount of findings have reported no difference in the limbic system, which might be a result of some potential moderators (i.e. the effect of illness severity, gender and medication effects) [8]. Further longitudinal work in larger samples is required to draw firm conclusions.

## Conclusions

The present study investigated the resting-state FC of regions with abnormal gray matter volume as the seed ROI. Patients with TRD and TSD showed significant gray matter abnormalities in the right MTG and bilateral caudate. Patterns of resting state FC for the two structural abnormal regions were different among the TRD, TSD and healthy controls. Our data provided the evidence for the aberrances of FC in DMN and reward circuit in patients with TRD and TSD. Overall, our results may provide a valuable basis for future studies combining morphometric and functional data for a comprehensive understanding of the neural circuitry underlying MDD.
